# Generation of Talbot-like fields

**DOI:** 10.1038/s41598-021-95697-x

**Published:** 2021-08-10

**Authors:** Jorge A. Anaya-Contreras, Arturo Zúñiga-Segundo, David Sánchez-de-la-Llave, Héctor M. Moya-Cessa

**Affiliations:** 1 Instituto Politécnico Nacional, Departamento de Física, ESFM, Edificio 9 Unidad Profesional Adolfo López Mateos, CP 07738 CDMX, Mexico; 2 Instituto Nacional de Astrofísica Óptica y Electrónica, Calle Luis Enrique Erro No. 1, 72840 Santa María Tonantzintla, Pue. Mexico

**Keywords:** Optics and photonics, Physics

## Abstract

We present an integral of diffraction based on particular eigenfunctions of the Laplacian in two dimensions. We show how to propagate some fields, in particular a Bessel field, a superposition of Airy beams, both over the square root of the radial coordinate, and show how to construct a field that reproduces itself periodically in propagation, i.e., a field that renders the Talbot effect. Additionally, it is shown that the superposition of Airy beams produces self-focusing.

## Introduction

In recent years there has been much interest in the propagation of light in free space where it has been shown that light not only propagates in straight lines but there are beams that also bend while propagating, such as the Airy beams^1–15^, which also present weak diffraction, i.e. they remain propagation invariant for distances that are much longer than the usual diffraction length of Gaussian beams with the same beamwidth^16^, self-healing, i.e. they regenerate themselves when a part of the beam is obstructed^17^, and abrupt autofocusing^18,19^, i.e. their maximum intensity remains constant while propagating and close to a particular point they autofocus increasing its maximum intensity by orders of magnitude. All of the above mentioned properties are very suitable for applications in medicine, several experimental settings where a sudden ignition is required for nonlinear processes, energy delivery on a remote target, imaging, particle manipulation, and material processing, to name just a few^18,20^.

Another interesting effect is the so called Talbot (or self-imaging) effect. The phenomenon was first observed by H. F. Talbot in 1836^21^. It is widely known for coherent monochromatic periodic fields in the Fresnel diffraction regime. Without the aid of lenses or any other optical element, the periodic field intensity repeats itself in planes located at multiples of the Talbot distance, defined by $\frac{2d^2}{l}$, where d is the period of the field and *l* is its wavelength. The field intensities located in planes between the Talbot distance maintain a periodic structure, although not necessarily with the same period. In this work, we present the less known case of nonperiodic objects, for which Montgomery^22–25^ has established the necessary and sufficient conditions for the self-imaging effect to take place, *i.e.*, the object Fourier spectrum must lie on the circles of a Fresnel zone plate. The Talbot effect has found applications not only in optics, but in other fields such as acoustics, electron microscopy, plasmonics, x-ray^26^, quantum state reconstruction of the electromagnetic field^27^ and Bose-Einstein condensates^26^. In optics, its main applications are related to image processing and synthesis, technology of optical elements, optical testing and optical metrology^28^.

In this contribution, by using eigenfunctions of the perpendicular Laplacian in polar coordinates, we propose a novel diffraction integral which we use to propagate some fields, namely, Bessel^29,30^ functions and superposition of Airy functions, both divided by the square root of the radial coordinate. As expected, the modified Bessel functions do not present propagation invariant properties whereas the superposition of modified Airy functions presents abrupt focusing^18,31–34^, a common effect in such superpositions. We also show that particular series of Bessel beams with integer or fractional order reproduce themselves during propagation, *i.e.*, giving rise to the Talbot effect. The integral of diffraction introduced in the manuscript may help in the search for structured light fields that maintain^35^, repeat their form or autofocus during propagation.

## Paraxial equation

We begin our analysis by recalling the paraxial equation, usually written as:

1 $$\begin{aligned} \nabla _{\perp }^2E+2ik \frac{\partial E}{\partial z}=0, \end{aligned}$$

whose solution is given by

2 $$\begin{aligned} E(x,y,z)=\exp \left[ i\frac{z}{2k}\nabla _{\perp }^2\right] E(x,y,0), \end{aligned}$$

where $\nabla _{\perp }^2$ is the Laplacian that in Cartesian coordinates can be expressed as

3 $$\begin{aligned} \nabla _{\perp }^2=\frac{\partial ^2}{\partial x^2}+\frac{\partial ^2}{\partial y^2}. \end{aligned}$$

In order to obtain the commonly used diffraction integral from (2), we first define the *operators* $D_x=\frac{\partial }{\partial x }$ and $D_y=\frac{\partial }{\partial y}$, therefore we may write the propagated field as

4 $$\begin{aligned} E(x,y,z)=\exp \left[ i\frac{z}{2k}D_x^2\right] \exp \left[ i\frac{z}{2k}D_y^2\right] E(x,y,0). \end{aligned}$$

Then we may write *E*(*x*, *y*, 0) in terms of its two-dimensional Fourier transform, *i.e.*,

5 $$\begin{aligned} E(x,y,0)=\frac{1}{2\pi }\int _{-\infty }^{\infty } \int _{-\infty }^{\infty } U(u,v)e^{iux}e^{ivy}dudv. \end{aligned}$$

such that we obtain

6 $$\begin{aligned} E(x,y,z)=\frac{1}{2\pi }\int _{-\infty }^{\infty } \int _{-\infty }^{\infty } U(u,v)e^{-i\frac{zu^2}{2k}}e^{-i\frac{zv^2}{2k}}e^{iux}e^{ivy}dudv, \end{aligned}$$

where we have used the fact that $e^{iux}$ is an eigenfunction of the operator $D_x$, with eigenvalue given by *iu* (similar expressions are obtained for the *y* coordinate). In the following sections, we employ the concepts of eigenfunctions and eigenvaules to produce an integral of diffraction that may be easily used when the field to be propagated is divided by the square root of the radial coordinate. In particular, we exploit the fact that in polar coordinates we may find a set of eigenfunctions described by

7 $$\begin{aligned} \left( \frac{\partial ^2 }{\partial r^2}+\frac{1}{r}\frac{\partial }{\partial r}+\frac{1}{r^2}\frac{\partial ^2}{\partial \theta ^2}\right) \frac{\hbox {e}^{\pm i\alpha r}}{\sqrt{r}}e^{\pm i\frac{\theta }{2}}= -\alpha ^2 \frac{\hbox {e}^{\pm i\alpha r}}{\sqrt{r}}\hbox {e}^{\pm i\frac{\theta }{2}}, \end{aligned}$$

with eigenvalues given by $-\alpha ^2$.

### Proposed diffraction integral

If we consider the field at $z = 0$ given in the form

8 $$\begin{aligned} E(r,\theta ,0)=\frac{\hbox {e}^{i\theta /2}}{\sqrt{r}}\int _{-\infty }^{\infty } {\mathcal {E}}(\alpha )\hbox {e}^{ir\alpha }d\alpha \;, \end{aligned}$$

and follow the procedure employed to obtain Eq. (6), a diffraction integral can be readily written as (we set $k=1$) (In all calculations, by replacing $z\rightarrow \frac{z}{k}$, arbitrary k’s may be considered).

9 $$\begin{aligned} E(r,\theta ,z)=\frac{\hbox {e}^{i\theta /2}}{\sqrt{r}}\int _{-\infty }^{\infty } \hbox {e}^{{-}i\frac{z}{2}\alpha ^2}{\mathcal {E}} (\alpha )\hbox {e}^{ir\alpha }d\alpha , \end{aligned}$$

where, for simplicity, we have used the plus sign in Eq. (7), but when we consider superpoistions of Airy functions below, we will use both signs. We have also applied the property that a function of the operator $\nabla _{\perp }^2$ applied to the eigenfunction is simply the function of the eigenvalue times the eigenfunction, *i.e.,*

10 $$\begin{aligned} F(\nabla _{\perp }^2)\frac{\hbox {e}^{i\alpha r}}{\sqrt{r}}\hbox {e}^{\pm i\frac{\theta }{2}}= F(-\alpha ^2) \frac{\hbox {e}^{i\alpha r}}{\sqrt{r}}\hbox {e}^{\pm i\frac{\theta }{2}}. \end{aligned}$$

## Propagating a Bessel function

Let us consider the following field at $z=0$

11 $$\begin{aligned} E(r,\theta ,z=0)=\frac{J_n(ar)}{\sqrt{r}}\hbox {e}^{i\frac{\theta }{2}}, \qquad n\ge 1, \end{aligned}$$

where $J_n(x)$ is a Bessel function of order *n* and the case $n=0$ is not considered because it would produce a singular field. We write the Bessel function in terms of its integral representation

12 $$\begin{aligned} E(r,\theta ,z=0)=\frac{1}{2\pi }\int _{-\pi }^{\pi }\frac{\hbox {e}^{iar\sin u}}{\sqrt{r}}\hbox {e}^{i\frac{\theta }{2}}\hbox {e}^{-inu}du, \end{aligned}$$

such that, by applying the property described by Eq. (10), we obtain

13 $$\begin{aligned} E(r,\theta ,z)=\frac{\hbox {e}^{i\frac{\theta }{2}}}{2\pi \sqrt{r}} \int _{-\pi }^{\pi }\hbox {e}^{{-}i\frac{a^2z}{2}\sin ^2 u}\hbox {e}^{iar\sin u-inu}du. \end{aligned}$$

Hereafter, we show that the integral above is a so-called Generalized Bessel function. First, we rewrite it as

14 $$\begin{aligned} E(r,\theta ,z)=\frac{\hbox {e}^{{-}i\frac{a^2z}{4}} \hbox {e}^{i\frac{\theta }{2}}}{2\pi \sqrt{r}}\int _{-\pi }^{\pi } \hbox {e}^{i\frac{a^2z}{4}\cos 2 u}\hbox {e}^{iar\sin u-inu}du, \end{aligned}$$

and define $Z=a^2z/{4}$ and use a Taylor series for the cosine term argument exponential, yielding

15 $$\begin{aligned} E(r,\theta ,Z)=\frac{\hbox {e}^{{-}iZ} \hbox {e}^{i\frac{\theta }{2}}}{2\pi \sqrt{r}}\sum _{m=0}^{\infty } \frac{(iZ)^m}{2^mm!}\int _{-\pi }^{\pi }(\hbox {e}^{2iu}+\hbox {e}^{-2iu})^m \hbox {e}^{iar\sin u-inu}du, \end{aligned}$$

and developing the binomial inside the integral we obtain

16 $$\begin{aligned} E(r,\theta ,Z)=\frac{\hbox {e}^{{-}iZ} \hbox {e}^{i\frac{\theta }{2}}}{2\pi \sqrt{r}}\sum _{m=0}^{\infty } \frac{(iZ)^m}{2^mm!}\sum _{p=0}^m \left( \begin{array}{c} m\\ p \end{array}\right) \int _{-\pi }^{\pi }\hbox {e}^{2iu(m-2p)}\hbox {e}^{iar\sin u -inu}du. \end{aligned}$$

We extend the second sum to infinity as we would only add zeros to the sum and exchange the order of the sums, yielding

17 $$\begin{aligned} E(r,\theta ,Z)=\frac{\hbox {e}^{{-}iZ}\hbox {e}^{i \frac{\theta }{2}}}{2\pi \sqrt{r}}\sum _{p=0}^{\infty }\sum _{m=0}^{\infty } \frac{(iZ)^m}{2^mp!(m-p)!} \int _{-\pi }^{\pi }\hbox {e}^{2iu(m-2p)}\hbox {e}^{iar\sin u-inu}du, \end{aligned}$$

and start the sum that runs on *m* at $m=m$ (as for $m<k$ the terms added are zero), *i.e.*,

18 $$\begin{aligned} E(r,\theta ,Z)=\frac{\hbox {e}^{{-}iZ}\hbox {e}^{i \frac{\theta }{2}}}{2\pi \sqrt{r}}\sum _{p=0}^{\infty }\sum _{m=p}^{\infty } \frac{(iZ)^m}{2^mp!(m-p)!} \int _{-\pi }^{\pi }\hbox {e}^{2iu(m-2p)}\hbox {e}^{iar\sin u-inu}du. \end{aligned}$$

By letting $j=m-p$ we obtain

19 $$\begin{aligned} E(r,\theta ,Z)=\frac{\hbox {e}^{{-}iZ}\hbox {e}^{i \frac{\theta }{2}}}{2\pi \sqrt{r}}\sum _{p=0}^{\infty }\sum _{j=0}^{\infty } \frac{(iZ)^{j+p}}{2^{j+p}p!j!}\int _{-\pi }^{\pi }\hbox {e}^{2iu(j-p)} \hbox {e}^{iar\sin u-inu}du, \end{aligned}$$

that, by using the integral representation of Bessel functions, we may write

20 $$\begin{aligned} E(r,\theta ,Z)=\frac{\hbox {e}^{{-}iZ}\hbox {e}^{i\frac{\theta }{2}} }{\sqrt{r}}\sum _{p=0}^{\infty }\sum _{j=0}^{\infty }\frac{(iZ)^{j+p}}{2^{j+p}p!j!} J_{n+2(p-j)}(ar). \end{aligned}$$

By letting $s=p-j$ we obtain

21 $$\begin{aligned} E(r,\theta ,Z)=\frac{\hbox {e}^{{-}iZ} \hbox {e}^{i\frac{\theta }{2}}}{\sqrt{r}}\sum _{s=-\infty }^{\infty }J_{n+2s} (ar)\sum _{j=0}^{\infty }\frac{(iZ)^{2j+s}}{2^{2j+s}(j+s)!j!}, \end{aligned}$$

where we have extended the sum on *s* to minus infinity as we simply add zeros.

Finally, the last equation can be rewritten as a sum of the products of two Bessel functions of different order, *i.e.*,

22 $$\begin{aligned} E(r,\theta ,Z)=\frac{\hbox {e}^{{-}iZ}\hbox {e}^{i\frac{\theta }{2}}}{\sqrt{r}}\sum _{s=-\infty }^{\infty }i^sJ_{n+2s}(ar)J_s({Z}), \end{aligned}$$

the so-called Generalized Bessel functions studied by Dattoli et al.^30,36^ and Eichelkraut^37^. By using that generalized Bessel functions, given by the expression ${\mathcal {J}}_n(r,z;g)=\sum _{s=-\infty }^{\infty }g^sJ_{n-2s}(r)J_s(z)$, we write the propagated field as

23 $$\begin{aligned} E(r,\theta ,Z)=\frac{\hbox {e}^{{-}iZ}\hbox {e}^{i\frac{\theta }{2}}}{\sqrt{r}}{\mathcal {J}}_n(ar,{Z};i). \end{aligned}$$

**Figure 1:**
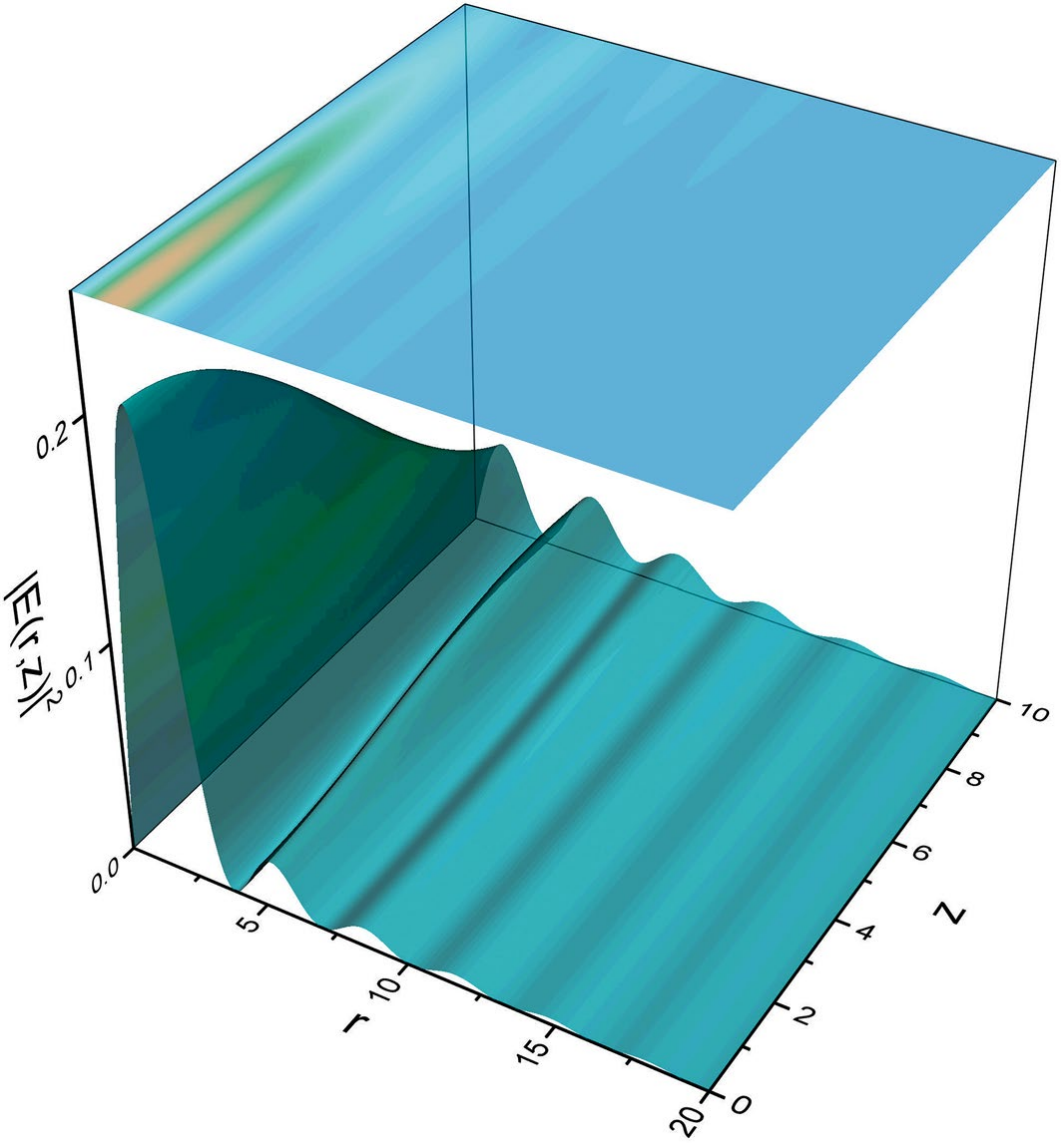
Intensity field distribution $\vert E(r,z)\vert ^2$ obtained from the initial state given in Eq. (11) with $n=1$ and $a=1$ (figure made with OriginPro 9.0. Available from https://www.originlab.com).

In Fig. 1 we plot the field intensity for an initial Bessel function of order $n=1$ as a function of the propagation distance *Z* and the radial coordinate. It may be observed that there is an energy redistribution from the central rings towards the outer rings as the field propagates, nevertheless, an overall intensity decrease also exists.

## Propagating a superposition of airy functions

We now study the propagation of a superposition of airy functions^2,6^. Its field distribution at $z=0$ is given by

24 $$\begin{aligned} E(r,\theta ,z=0)=\frac{\hbox {e}^{i\frac{\theta }{2}}}{2\pi \sqrt{r}} \left[ \int _{-\infty }^{\infty }\hbox {e}^{i\left( \frac{t^{3}}{3} +rt\right) }\right. dt - \left. \int _{-\infty }^{\infty }\hbox {e}^{i\left( \frac{t^{3}}{3}-rt \right) }dt\right] , \end{aligned}$$

where we have written the Airy function in its integral representation. By applying the integral of diffraction given by Eq. (9) we obtain

25 $$\begin{aligned} E(r,\theta ,z)=\frac{\hbox {e}^{i\frac{\theta }{2}}}{2\pi \sqrt{r}} \left[ \int _{-\infty }^{\infty }\hbox {e}^{i\left( \frac{t^{3}}{3}+rt\right) } \hbox {e}^{{-}i\frac{zt^2}{2}}dt\right. -\left. \int _{-\infty }^{\infty }\hbox {e}^{i\left( \frac{t^{3}}{3}-rt \right) }\hbox {e}^{{-}i\frac{zt^2}{2}}dt\right] . \end{aligned}$$

By changing variables in the integrals above, we may rewrite them as

26 $$\begin{aligned} E(r,\theta ,z)=\frac{\hbox {e}^{{-}i\frac{z^3}{12}}\hbox {e}^{i\frac{\theta }{2}}}{2\pi \sqrt{r}}\left[ \hbox {e}^{i\frac{rz}{2}}\int _{-\infty }^{\infty } \hbox {e}^{i\left( \frac{t^{3}}{3}+\left[ r{-} \frac{z^2}{4}\right] t\right) }dt\right. -\left. \hbox {e}^{{-}i\frac{rz}{2}}\int _{-\infty }^{\infty }\hbox {e}^{i\left( \frac{t^{3}}{3}-\left[ r{+}\frac{z^2}{4}\right] t\right) }dt\right] , \end{aligned}$$

that finally yields the following superposition of Airy functions

27 $$\begin{aligned} E(r,\theta ,z)=\frac{\hbox {e}^{{-}i\frac{z^3}{12}} \hbox {e}^{i\frac{\theta }{2}}}{\sqrt{r}} \left( \hbox {e}^{{+}i\frac{rz}{2}} \hbox {Ai}\left[ r{-}\frac{z^2}{4}\right] \right. -\left. \hbox {e}^{{-}i\frac{rz}{2}}\hbox {Ai}\left[ -r{-} \frac{z^2}{4}\right] \right) . \end{aligned}$$

**Figure 2:**
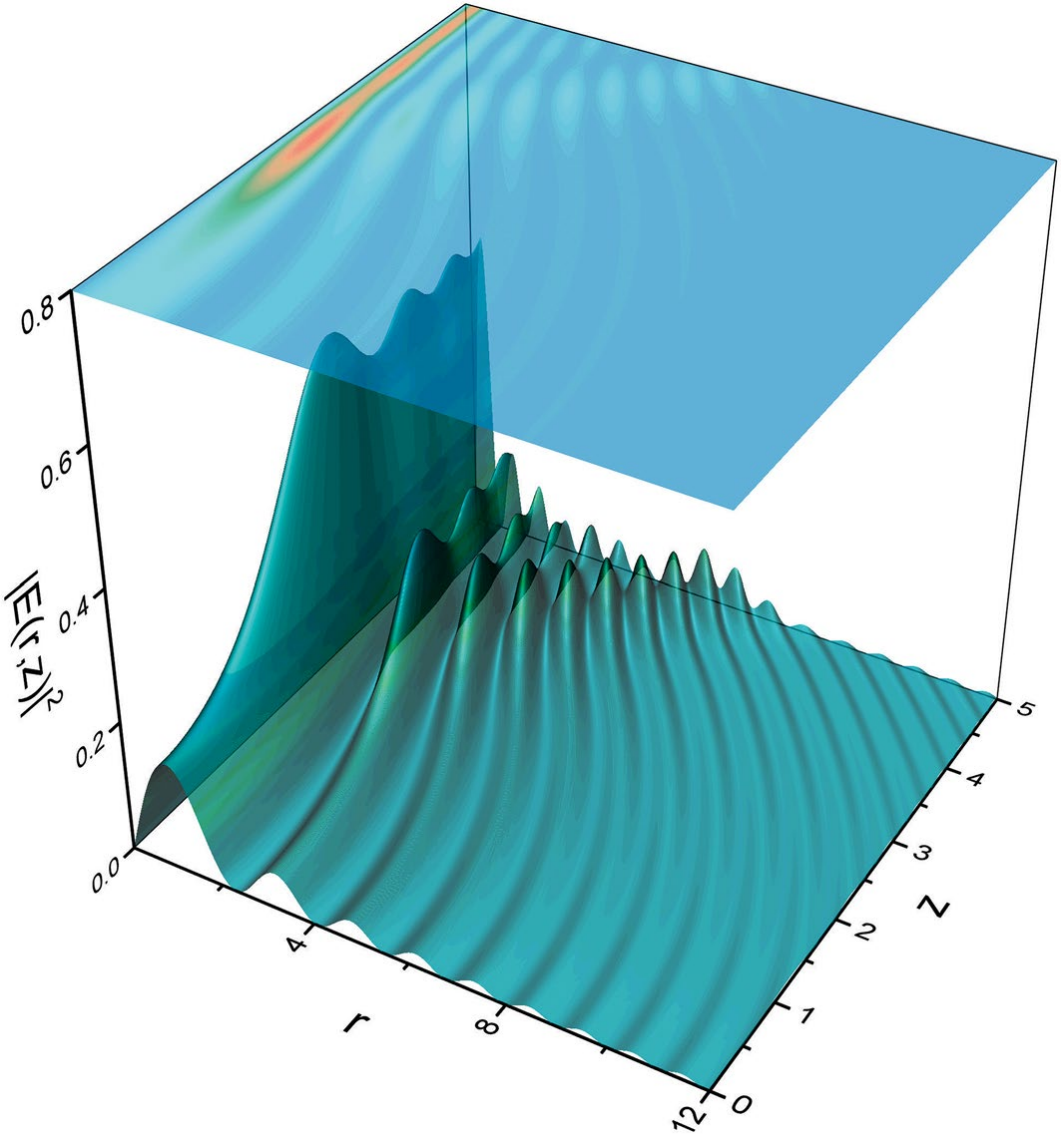
Intensity field distribution $\vert E(r,z)\vert ^2$ obtained from the initial state given by Eq. (24) (figure made with OriginPro 9.0. Available from https://www.originlab.com).

We plot the propagated field intensity in Fig. 2 where the abrupt focusing observed may be attributed to the superposition of the Airy functions. There is one Airy function whose main contribution would be in the negative part of the axis, and would bend towards the right. However, as *r* is always positive, it does not have enough weight to produce an effect. On the other hand, the Airy function whose main contribution is on the positive part, dominates the propagation and bends towards the left. Although there is no medium, the focusing may be explained by the fact that the Airy function *produces* an effective index of refraction (the so-called Bohm potential in quantum mechanics)^38,39^ that gives rise to such behaviour.

## Talbot effect for the superposition of Bessel functions of order $\frac{1}{2}$

**Figure 3:**
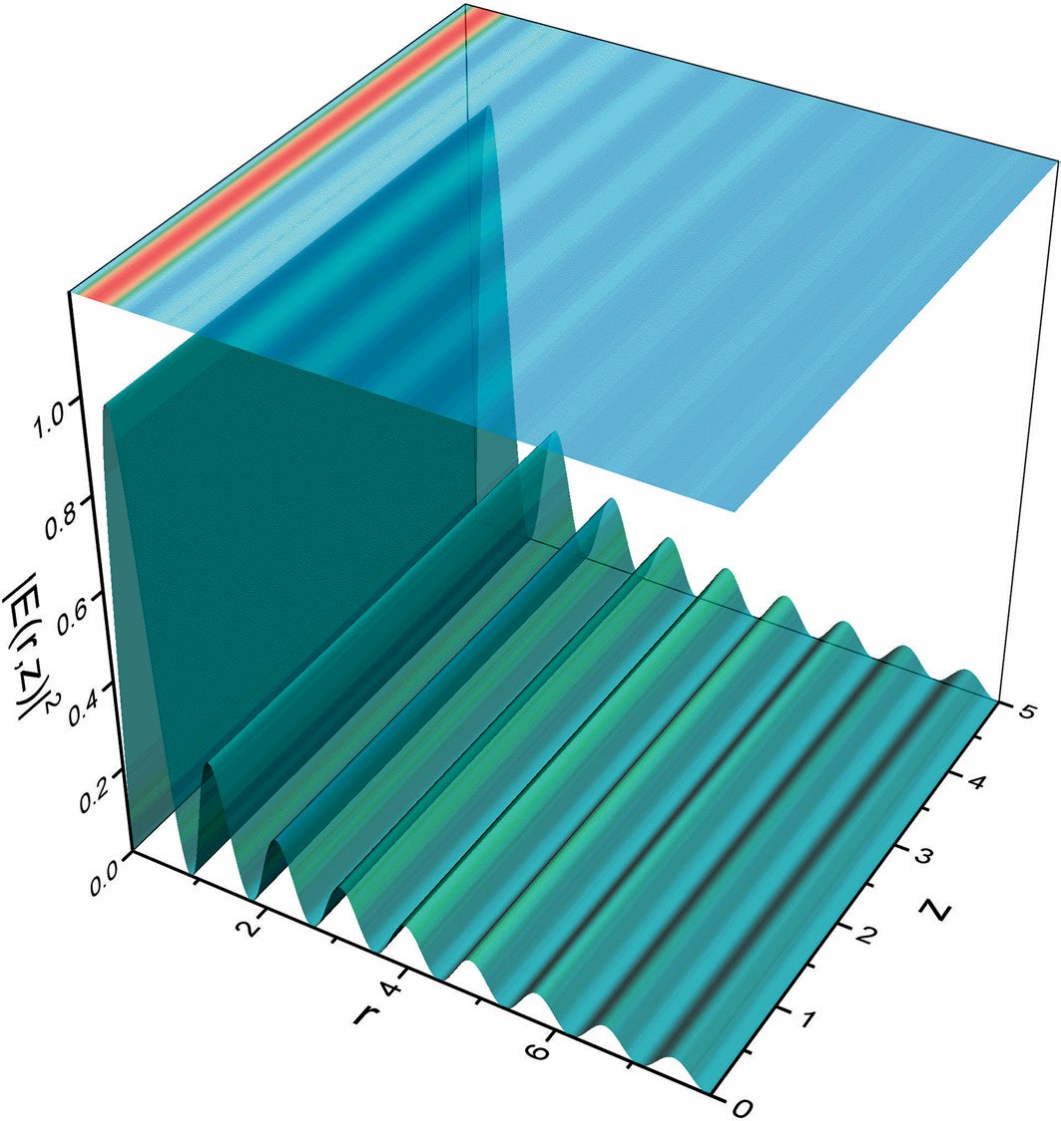
Normalized field intensity distribution $\vert E(r,z)\vert ^2$ obtained from the initial state (28) (figure made with OriginPro 9.0. Available from https://www.originlab.com).

We can superimpose the eigenfunctions described by Eq. (7) with the same eigenvalue to find another eigenfunction, a beam of the form $\frac{\sin {br}}{\sqrt{r}}$, which takes us to a Bessel function of order one half, described by

28 $$\begin{aligned} E(r,\theta ,z=0)=J_{\frac{1}{2}}(br)\hbox {e}^{i\frac{\theta }{2}}, \end{aligned}$$

that is indeed, a diffraction-free beam. A plot of its intensity as a function of the radial coordinate and the propagation distance z is depicted in Fig. 3.

It is straight forward to show that a superposition of them, namely

29 $$\begin{aligned} E(r,\theta ,z=0)=\hbox {e}^{i\frac{\theta }{2}}\sum _{m=1}^N c_mJ_{\frac{1}{2}}\left( 2\sqrt{{m}\pi }r\right) , \end{aligned}$$

where *N* indicates the number of components that the field has (at $z=0$) and $c_m$ is a weight that is written in order to have the most arbitrary field possible, propagates as

30 $$\begin{aligned} E(r,\theta ,z)=\hbox {e}^{i\frac{\theta }{2}}\sum _{m=1}^Ne^{-i2{m}{z}\pi } c_mJ_{\frac{1}{2}}\left( 2\sqrt{{m}\pi }r\right) . \end{aligned}$$

We note that the field described by the last equation presents the interesting property of repeating itself periodically at values of $z=n \, (n=1,2,3,...)$, this is:

31 $$\begin{aligned} E(r,\theta ,z=n)=\hbox {e}^{i\frac{\theta }{2}}\sum _{m=1}^Ne^{-i2mn\pi } c_m J_{\frac{1}{2}}\left( 2\sqrt{{m}\pi }r\right) =E(r,\theta ,z=0). \end{aligned}$$

**Figure 4:**
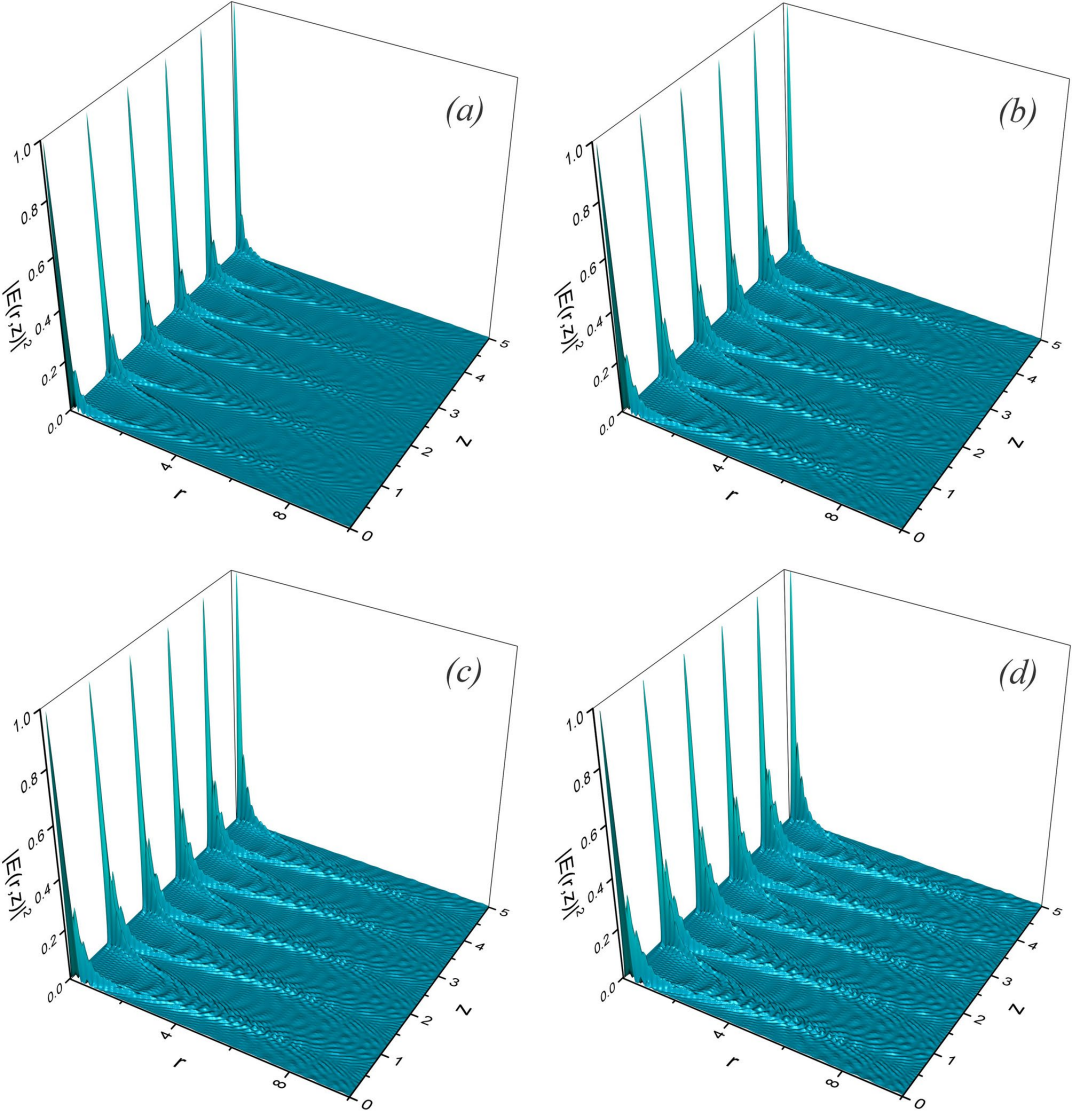
Normalized Field intensity distribution $\vert E(r,z)\vert ^2$ obtained from the non-periodic initial state given by Eq. (35) with $N=20$ and $c_m=1$, for (**a**) $\nu =1/2$, (**b**) $\nu =1$, (**c**) $\nu =3/2$, (**d**) $\nu =2$ (figure made with OriginPro 9.0. Available from https://www.originlab.com).

The self-imaging effect can be seen clearly in Fig. 4a, where a plot of the normalized field intensity is shown, for $c_m=1$ and $N=10$, as a function of the radial coordinate and the propagation distance *z*. We remark the fact that the propagated field is not periodic, nevertheless it fullfills the conditions stablished by Montgomery^22^ for the Talbot effect to take place.

### Generalization of the Talbot effect to a superposition of Bessel functions of any order

It is well-known that Bessel functions (of integer or fractional order) obey the differential equation^40^

32 $$\begin{aligned} \frac{d^2J_{\nu }(\beta r)}{dr^2}+\frac{1}{r}\frac{dJ_{\nu }(\beta r)}{dr}+\left( \beta ^2-\frac{\nu ^2}{r^2}\right) J_{\nu }(\beta r)=0, \end{aligned}$$

which, if multiplied by $\hbox {e}^{i\nu \theta }$, may be rewritten as

33 $$\begin{aligned} \left( \frac{d^2}{dr^2}+\frac{1}{r}\frac{d}{dr}+\frac{1}{r^2}\frac{d^2}{d\theta ^2}\right) J_{\nu }(\beta r)\hbox {e}^{i\nu \theta }=-\beta ^2J_{\nu }(\beta r)\hbox {e}^{i\nu \theta }, \end{aligned}$$

or

34 $$\begin{aligned} \nabla _{\perp }^2J_{\nu }(\beta r)\hbox {e}^{i\nu \theta }=-\beta ^2J_{\nu }(\beta r)\hbox {e}^{i\nu \theta }, \end{aligned}$$

making the functions $J_{\nu }(\beta r)\hbox {e}^{i\nu \theta }$ eigenfunctions (with eigenvalue $-\beta ^2$) of the Laplacian in polar coordinates and therefore becoming propagation invariant fields^29^. Therefore a field at $z=0$ given by

35 $$\begin{aligned} E(r,\theta ,z=0)=\hbox {e}^{i\nu \theta }\sum _{m=1}^N c_mJ_{\nu }\left( 2\sqrt{{m}\pi }r\right) , \end{aligned}$$

propagates as

36 $$\begin{aligned} E(r,\theta ,z)=\hbox {e}^{i\nu \theta }\sum _{m=1}^N\hbox {e}^{-i2{m}{z}\pi } c_mJ_{\nu }\left( 2\sqrt{{m}\pi }r\right) , \end{aligned}$$

yielding Eq. (30) for $\nu =1/2$. Therefore, the field at $z=0$ reproduces itself periodically at propagation distances given by $z=n \, (n=1,2,3, ...)$, *i.e.*,

37 $$\begin{aligned} E(r,\theta ,z=n)=\hbox {e}^{i\nu \theta }\sum _{m=1}^N\hbox {e}^{-i2{m}n\pi } c_m J_{\nu }\left( 2\sqrt{{m}\pi }r\right) = E(r,\theta ,z=0). \end{aligned}$$

Plots of the normalized field intensity are shown in Fig. 4b–d for v=1, 3/2, and 2, respectively. The values of $c_m$ and *N* are 1 and 20, respectively. All non-periodic fields shown in Fig. 4 clearly exhibit the Talbot effect.

## Conclusions

We have shown that by properly writing a field at $z=0$ we may propagate it by using a novel diffraction integral that we introduced in this manuscript. We have shown how to propagate Bessel and a superposition of Airy beams (over the square root of the radial coordinate) and have shown that a series of Bessel functions that may have integer or fractional order and with proper parameters reproduces itself during propagation, therefore producing the Talbot effect. We have shown self focusing of the superposition of Airy beams that may be explained by the existence of an *effective* index of refraction related to the Bohm potential.
